# Identifying the Genetic Distance Threshold for Entiminae (Coleoptera: Curculionidae) Species Delimitation via COI Barcodes

**DOI:** 10.3390/insects13030261

**Published:** 2022-03-05

**Authors:** Zhuo Ma, Jinliang Ren, Runzhi Zhang

**Affiliations:** 1 Key Laboratory of Zoological Systematics and Evolution, Institute of Zoology, Chinese Academy of Sciences, Beijing 100101, China; mazhuo@ioz.ac.cn (Z.M.); renjinliang@ioz.ac.cn (J.R.); 2 College of Life Science, University of Chinese Academy of Sciences, Beijing 100049, China

**Keywords:** DNA barcoding, Entiminae, genetic distance, species delimitation

## Abstract

**Simple Summary:**

Over 12,000 species of the subfamily Entiminae (Coleoptera: Curculionidae) have been described worldwide, but there has yet to be realization of the potential for DNA barcodes to assist in species-level identification. Here, we analyzed the variation in intra- and interspecific genetic distance of 621 public- and 27 self-determined sequences, to determine parameters for species identification of Entiminae. Our study found no universal barcoding gap at the subfamily level, although a genetic distance threshold of 9.18% can delimit more than 88% of Entiminae species. We also inferred additional empirical threshold values for 14 genera (species > 2) that can delimit congeneric species.

**Abstract:**

The subfamily Entiminae is the largest group in the family Curculionidae, and it has long represented a challenge in traditional and molecular classification. Here, we analyzed intra- and interspecific genetic distances of 621 public COI barcode sequences (658bp) from 39 genera and 110 species of Entiminae, to determine parameters most congruent in retaining established species. We found that the mean intraspecific genetic distance (3.07%) was much smaller than the mean interspecific one (21.96%), but there is a wide range of overlap between intra- and interspecific genetic distances (0.77–18.01%), indicating that there is no consistent, universal barcoding gap. Specifically, DNA barcoding gap analysis for morphospecies revealed that 102 of 110 morphospecies had barcoding gaps, and 9.18% was the optimum threshold of genetic distances for 97 species delimitation. We further confirmed this threshold with barcodes from 27 morphologically identified specimens (including 21 newly reported barcodes) sequenced from five genera and seven species. We also identified thresholds to delimit congeneric species within 14 selected genera (species > 2), which varied from 7.42% (*Trichalophus*) to 13.48% (*Barypeithes*). We herein present optimal parameters for species identification in the Entiminae. Our study suggests that despite no universal genetic distance threshold value in subfamily Entiminae, 9.18% is optimal for most species. We recommend a wider sampling of geographic populations to better account for intraspecific distance variation, and that genetic distance thresholds for species delimitation should be refined at the genus level.

## 1. Introduction

Weevils (Coleoptera: Curculionoidea) are one of the most diverse and ubiquitous insect groups in Coleoptera, with more than 62,000 identified species (5800 genera) [1,2], constituting nearly 15% of described Coleoptera [3]. Entiminae is the largest subfamily in Curculionidae, with more than 12,000 described species within 1280 genera, including many key agricultural pests [2,4]. Most Entiminae are polyphagous, with adults feeding on leaves and young shoots of trees and shrubs, while the larvae feed on roots [3]. As pests, their effective management requires rapid diagnostic tools. Morphological characteristics are often used for species description and identification. Characters such as the deciduous mandibular cusps, corbels on the hind tibiae also occur in Brachycerinae (Curculionoidea: Curculionidae) [3], and many taxa often include cryptic species complexes [5,6]. The morphological differences between closely related species can be so small that even professional taxonomists struggle with identification [4]. Several factors, including polymorphic species [7], sexual dimorphism [8], and immature stages (egg, larva, pupa), may further impede this process. Therefore, molecular tools combined with morphological traits appear most promising for specimen identification.

Early molecular methods for species delimitation used alloenzyme variability and gene flow (based on Wright’s *F_st_* statistics), allele frequency, and nuclear gene codominance [9,10,11,12,13]. Currently, the most widely applied method is DNA barcoding, the use of a standard 650 bp fragment of mitochondrial gene cytochrome oxidase I (COI), and has been proven a useful tool in Entiminae taxonomy [4,14,15]. Barcodes have been used to delimit species when other approaches have failed [16]. In DNA barcoding, taxonomic names are assigned to query sequences after comparing their sequences with those of reference databases. Although it is more accurate to use multiple loci for species delimitation analyses [17], single-locus species delimitation method is still widely applied in DNA barcoding studies of bacteria, fungi and invertebrates [18,19]. Over the last 17 years, 9395 Entiminae COI–5′ sequences from 534 species were available in the Barcode of Life Data Systems (BOLD, http://www.boldsystems.org/, 15 October 2020), providing valuable resources for species delimitation in this subfamily.

The reliability of DNA barcoding relies on the level of intraspecific variation in sequences relative to interspecific (barcoding gap) [20]. Independent of the presence or width of the barcoding gap, a distance threshold needs to be selected which represents the value at which molecular clusters are most similar to assigned taxonomic species, and thus the value at which a query sequence can be confidently assigned to species. Thresholds are not necessarily the same across taxa for COI. Hebert et al. proposed 3% genetic distance of COI as the threshold for lepidopterans [21]. However, in many cases, using a fixed genetic threshold to distinguish taxa with different evolutionary histories may overestimate or underestimate species diversity [20,22,23,24,25]. Instead of depending on fixed thresholds, it is better to directly generate an optimized threshold from the data [20,26]. We also need to reassess these threshold values when taxonomic knowledge is updated and new sequences are generated, to improve its reliability and practical applications [18].

In this study, we tested all Entiminae COI–5′ sequences from BOLD and self-determined sequences to (1) provide an overview of sequences and species information available in BOLD; (2) test the existence of a universal barcoding gap useable at subfamily level; (3) if no universal threshold exists, establish a threshold value to delimit most Entiminae species (4) set up threshold values for congeneric species where feasible.

## 2. Materials and Methods

### 2.1. Publicly Available Data

We downloaded 9395 COI–5′ sequences of 534 Entiminae species (162 genera) collected from 50 countries (up to October 2020, Figure 1) from the BOLD database (Table S1). To refine our dataset, we filtered and removed sequences (1) unidentified to the species level (e.g., “*Gymnopholus* sp.”), (2) without GenBank accession numbers, (3) with degenerate bases (such as K, M, R, S, W, Y, N, X), and (4) shorter than 658bp. Multiple sequence alignment and pruning were performed using MAFFT ver. 7 (with default parameters) and MEGA ver. 7, respectively [27,28], to generate a standard 658 bp barcode near the 5′ end of the COI. For each species, the duplicate sequences were removed. The species with only one sequence were filtered out because the intraspecific genetic distance (intra-GD) was inaccessible. All sequences were translated into amino acids to check and avoid stop-codons via MEGA ver. 7 [28]. Some synonymous names involving 39 sequences of ten species were manually revised according to the scientific name provided by the website https://www.uniprot.org/, 30 October 2020 (Table S2).

### 2.2. Sample Collection and Identification

**Figure 1:**
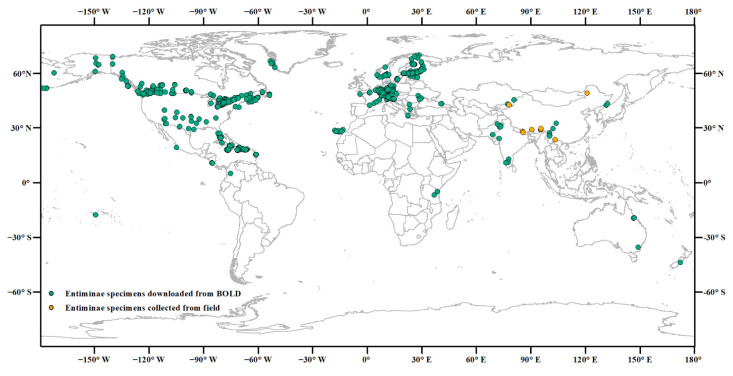
Sampling localities of Entiminae specimens for which COI barcodes were analyzed. The green and orange dots represent the samples downloaded from the BOLD database, and newly collected, respectively.

In 2018 and 2019, 54 specimens of Entiminae were collected in ten locations in three of China’s provinces including Inner Mongolia, Tibet, and Shandong (48 specimens belonging to six species) and in one location of Kyrgyzstan (6 specimens belonging to one species) (Figure 1, Table S4). Sampling methods included net-sweeping during the day and visually searching during the night. The latitude and longitude were recorded. Specimens were fixed in 95% ethanol at −20 °C until use. All collected specimens were morphologically identified by taxonomist Dr Li Ren [29,30].

### 2.3. DNA Extraction, Amplification and DNA Sequencing

We extracted DNA from a total of 54 specimens via DNeasy Blood & Tissue Kits (Qiagen, Germany). DNA was extracted from either 1, 3, 6 legs or the whole body, depending on the size of specimen, all voucher specimens were preserved at the Institute of Zoology, Chinese Academy of Sciences. PCR amplifications for COI sequences were conducted using the cocktail primers C_LepFolF and C_LepFolR (Table 1) [31]. If the amplification based on this primer pair failed, we used the amplification strategies provided by Hebert et al. for 307-bp and 407-bp fragments by universal primer sets (C_LepFolF + MLepR2 and MLepF1 + C_LepFolR, Table 1) [32,33]. The full length 658bp COI sequence was spliced during post hoc analysis. PCR reaction mixes (25 mL) contained 14 μL 2 × Taq PCR MasterMix (Tiangen Biotech Co., Ltd., Beijing, China), 1 μL of forward and reverse primer each (Sangon Biotech Co. Ltd., Shanghai, China), 2 μL total undiluted DNA template, and 7 μL dd H_2_O. PCR profile as follows: 94 °C for 2 min, first cycle set (5 repeats): 94 °C for 40 s, 45 °C for 40 s and 72 °C for 60 s. Second cycle set (35 repeats): 94 °C for 40 s, 51 °C for 40 s and 72 °C for 60 s, followed by elongation at 75 °C for 5 min. PCR products were visualized through 1% agarose gel electrophoresis in TAE buffer. Successful PCR products were sent for sequencing in the Beijing Genomics Institute (BGI, Shenzhen, China). The raw data were assembled and edited via SeqMan software (Lasergene software version 7.1) [34]. Then the same screening criteria (see Section 2.1) were used to filter the generated sequences.

### 2.4. Sequence Analysis

In this study, three datasets were generated: dataset I included species with unique sequences downloaded from BOLD; dataset II included species with unique sequences generated by ourselves; and dataset III was the combination of dataset I and II. All sequences from dataset III were inputted into MEGA ver. 7. We recorded the nucleotide composition, conserved sites, variable sites, parsimony–information site, and singleton sites [28].

The intra- and interspecific K2P genetic distances of datasets I, II and III were separately calculated using the sppDist function of the R package spider [35]. The frequency distribution of intra- and interspecific genetic distances was counted with an interval of 0.5%, and three histograms were drawn based on statistical results (x-axis: genetic distance; y-axis: frequency). The overall minimum, maximum, and average intra- and interspecific genetic distances were also calculated for three datasets.

The maximum intraspecific genetic distance (intra-GD) and minimum interspecific genetic distance (inter-GD) of dataset I, II, III were calculated using the maxInDist and nonConDist function of spider R package [35]. Minimum distances among species and maximum distances within species, rather than the average distance, were critical for credible identification [36]. To confirm the existence of barcoding gaps, three scatter plots were drawn based on these datasets (*x*-axis: maximum intra-GD; y-axis: minimum inter-GD). Point above 1:1 slope suggested a barcoding gap for that species [37].

For dataset I, the localMinima function in spider R package was used to calculate the possible thresholds of genetic distances for species delimitation [35]. For each candidate threshold, we recorded the number of species whose barcoding gap contains this threshold value (the threshold is in the interval between the maximum intra-GD and the minimum inter-GD), and the threshold with largest number of species was selected as the optimum one. We then compared the molecular delimitation efficiency between the selected thresholds from dataset I and the threshold (2.20%) used by BINs (Barcode Index Numbers) system [38], by examining the number of species whose barcoding gap contains the threshold value in dataset II and dataset III. Furthermore, in dataset III, we only analyzed the 14 genera with at least two species to ensure intra- and interspecific comparisons. For each genus, the maximum intra-GD and minimum inter-GD were calculated using the maxInDist and nonConDist function of spider R package [35], we also calculated the genetic distance thresholds using the localMinima function [35]. Then the same step was implemented to determine the optimum threshold for species delimitation within genera.

## 3. Results

### 3.1. Downloading Data

After duplicate removal, 2 to 36 sequences (mean = 6) were obtained for each species, with *Diaprepes abbreviates* having the highest number. The number of species in each genus ranged from 1 to 22 (mean = 3), with *Otiorhynchus* containing the most. A final dataset used for subsequent analyses included 621 COI–5′ sequences representing 39 genera and 110 species of Entiminae collected from 23 countries. Collection localities of 14 specimens were unavailable. Relevant data (Species names, GenBank accession codes, BOLD Process ID, BINs, Collection information, and COI–5′ sequence) are available in Supplementary material Table S3.

### 3.2. Sequencing Data

We successfully amplified targeted fragments of all 54 specimens using a combination of multiple primer strategies. The success rate of amplification is given in Table 2. After assembling overlapping fragments for mini-barcodes (of 307 bp and 407 bp, Table 2), we only failed to obtain a full length of 658 bp from three specimens. The final dataset II used for subsequent analyses included 27 unique COI–5′ barcode sequences from seven morphospecies (5 genera), in which two morphospecies had been identified to genus level and five ones to species level (Table S5). The 27 sequences were submitted to GenBank (Accession nos. OK575948–OK575974), including 21 novel DNA barcodes for five species (*Leptomias viridicantis*, *Leptomias huangi*, *Leptomias acutus*, *Sitona* sp., *Sympiezomias* sp.).

### 3.3. Nucleotide Composition

We obtained 648 COI sequences with a length of 658 base pairs (bp) from 41 genera and 117 species, and then integrated them into dataset III. No insertions, deletions, stop-codons, or sequencing errors were detected in any sequences. There were overall 403 variable sites, of which 378 were parsimony informative. Most variable sites occurred in the third codon position. The average nucleotide compositions of the COI sequences were T = 35.4%, C = 18.9%, A = 29.6%, and G = 16.1%. The sequences were heavily AT-biased (65.0%), especially in the third codon position (82.5%) (Table 3).

### 3.4. Genetic Distance

**Figure 2:**
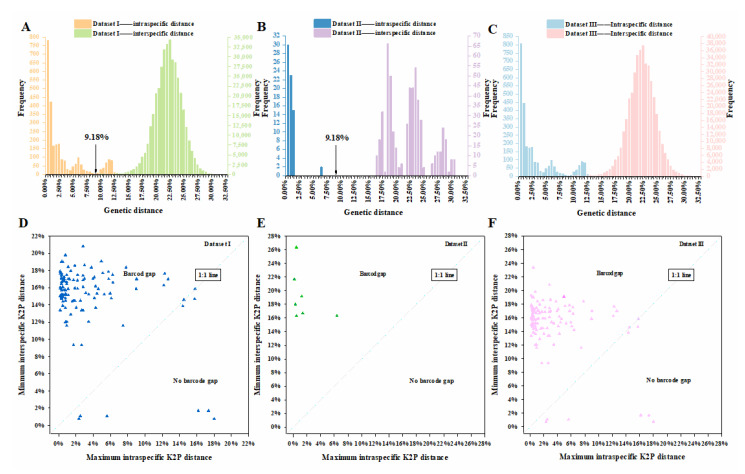
Intra- and inter-GD histograms of dataset I (**A**), dataset II (**B**), and dataset III (**C**). The marked 9.18% indicates a suggested delimitation genetic distance threshold for most Entiminae; (**D**–**F**) Scatter plot of the max intra-GD versus min inter-GD for the corresponding dataset above. Each triangle represents a species. The dot falling below the 1:1 slope indicating the absence of a “barcode gap”.

For the downloaded sequences in dataset I, the intra-GD ranged from 0.15% to 18.01% (mean = 3.07%), while the inter-GD ranged from 0.77% to 31.60% (mean = 21.96%) (Figure 2A; Table S6). Although the mean inter-GD was 7-fold higher than the mean intra-GD, the overlap between inter- and intra-GD (0.77–18.01%) indicated the absence of the barcoding gap (Figure 2A). In the 110 species, intra-GD of 59 species were <2.00% and the maximum intra-GD (18.01%) was detected in *Polydrusus impressifrons* (Table S7).

For the generated sequences in dataset II, the intra-GD ranged from 0.15% to 6.39% (mean = 0.81%), and the inter-GD ranged from 16.33% to 30.18% (mean = 22.05%) (Figure 2B; Table S6). The ranges of both intra- and inter-GD were contained by the corresponding ranges in dataset I (Figure 2B). The maximum intra-GD was much less than the minimum inter-GD, indicating the presence of a barcoding gap. In the seven species, six had intra-GD < 2.00% and the maximum intra-GD (6.39%) was detected in *Leptomias acutus* (Table S8).

For the integrated dataset III, the intra-GD ranged from 0.15% to 18.01%, and the inter-GD ranged from 0.77% to 32.78% (Figure 2C). The mean intra-GD (3.02%) was much lower than the mean inter-GD (22.02%) (Table S6).

### 3.5. Barcode Gap Analysis

In dataset I, the mean values of maximum intra-GD (0.15–18.01%) and minimum inter-GD (0.77–28.84%) were 3.33% and 15.03%, respectively (the latter was almost five times larger than the former; Table S7). A barcode gap was detected in 102 of 110 (92.7%) species, and 9.18% was an optimum threshold of genetic distance for the delimitation of 97 (88.2%) species (Figure 2D, Tables S6 and S7). However, only 63 species (57.3%) could be delimited with a genetic distance threshold of 2.20% (Tables S6 and S7).

In dataset II, the mean values of maximum intra-GD (0.15–6.39%) and minimum inter-GD (16.33–26.37%) were 1.48% and 19.22%, respectively (the latter was almost 13 times larger than the former; Table S8). A barcode gap was detected in all seven species (100.0%) (Figure 2E, Table S8). The optimum threshold of 9.18% in dataset I can also be used for delimitating the seven species in dataset II, whereas the threshold of 2.20% was only useful to delimit six species (Tables S6 and S8).

In dataset III, the mean values of maximum intra-GD (0.15–18.01%) and minimum inter-GD (0.77–23.39%) were 3.22% and 15.16%, respectively (the latter was almost five times higher than the former; Table S9). A barcode gap was detected in 109 of 117 (93.2%) species (Figure 2F, Table S9). We calculated the genetic distance threshold for species delimitation within 14 genera (with species more than two) and found the highest value in the genus *Barypeithes*: a threshold of 13.48% could delimit two (100%) species of *Barypeithes* (Table S10). We found the lowest value in the genus *Trichalophus*, where the threshold 7.42% could delimit two (100.0%) species of *Trichalophus* (Table S10). Distance thresholds in other genera were: 13.30% (*Otiorhynchus*), 10.16% (*Polydrusus*), 9.72% (*Brachyderes*), 9.66% (*Peritelus*), 9.39% (*Leptomias*), 9.21% (*Chlorophanus*), 9.15% (*Strophosoma*), 9.09% (*Sitona*), 8.59% (*Trachyphloeoides*), 8.09% (*Pachyrhinus*), 7.91% (*Trachyphloeus*), and 7.55% (*Phyllobius*) (Table S10).

## 4. Discussion

### 4.1. The Choice of Primers

Following delimitation, affirmative species identification will be necessary, and this relies on the sequencing of many barcodes. Choosing a universal primer with versatility and applicability is critical for the overall success of the endeavor [39]. LCO1490 + HCO2198 are the most widely used primers for amplifying insect mitochondrial CO1 barcodes, and the primer pair LepF1 + LepR1 are commonly used for amplification of hemipteran and lepidopteran barcodes [33,39,40,41]. However, these two primer pairs are rarely used in weevils, in part because the amplified sequences are usually of poor quality [41]. We used a combination of two strategies involving three fragments (658bp, 307bp, and 407bp) for amplification, by following the guidelines of the Canadian Centre for DNA Barcoding (CCDB) and the literature of Hernández-Triana et al. and Hendrich et al. [31,42]. This amplification strategy (307 bp + 407 bp) is often used for specimens in museum collections [32,39]. Our results suggest that this method is effective, and the success rate of amplification increases from 61.1% to 87.0% compared with only using the primer pair C_LepFolF + C_LepFolR.

**Figure 3:**
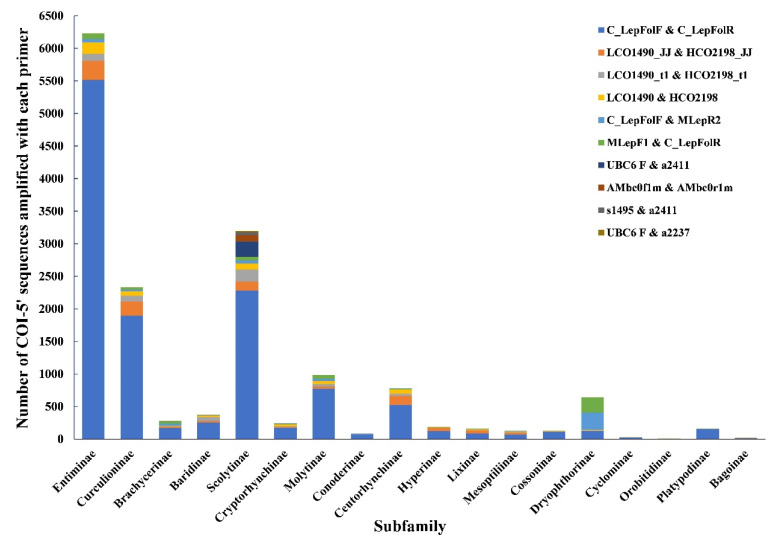
Primers sets used to amplify COI barcode sequences of different subfamilies in Curculionidae.

The primers for our first amplification, C_LepFolF and C_LepFolR, were chosen from the BOLD primer database and had been used to previously amplify 18,307 of 22,231 Curculionidae COI–5′ barcode sequences. For most subfamilies (17 of 18), e.g., Entiminae, Curculioninae, Scolytinae, Molytinae, Ceutorhynchinae, and so on, the most widely used primers are also C_LepFolF and C_LepFolR (Figure 3). Although there is a large database of primers in BOLD, beginners should carefully select primers according to taxa under study, with preliminary experiments including extra primers design maximizing the chances of success. For example, we found that the primers pairs UBC6 F + a2411, UBC6 F + a2237, s1495 + a2411, and AMbc0f1m + AMbc0r1m were mainly used to amplify the COI–5′ sequence of subfamily Scolytinae (Table S11, Figure 3), which could supplement LCO1490 + HCO2198 used by Albo et al. [41].

### 4.2. An Optimal Genetic Distance Threshold for Entiminae

In the ideal case, the interspecific divergence is larger than intraspecific, as this enables distinguishing species. Some studies have detected barcoding gaps, and some not [4,25,43,44,45,46]. Several factors including cryptic species, insufficient sampling, geographical isolation, erroneous taxonomic assignment, incomplete lineage sorting, and homoplasy can all cause exceptionally low inter-GD or high intra-GD, therefore causing the absence of a barcoding gap [20,36,46,47,48]. Therefore, a universal, optimum threshold value cannot be determined.

Different species possessing identical sequences is relatively common in insects [49]. For example, among coleopteran species (3531 species, 15,948 individuals) in central Europe, including representatives of Carabidae, Cerambycidae, Mordellidae, Mycetophagidae, Oedemeridae, Phalacridae, and Staphylinidae, Hendrich et al. found the inter-GD of 33 morphologically distinct species (155 specimens, involving 15 pairs and one triplet) was 0.0% [42]. Astrin et al. studied the species boundaries of 217 western Palaearctic Cryptorhynchinae (Coleoptera: Curculionidae) insects and found several closely related species had identical sequences (e.g., *Madeiracalles terminalis* + *Madeiracalles tolpis*, *Silvacalles instabilis* + *Silvacalles nubilosus*, *Dendroacalles ruteri + Dendroacalles fortunatus*, *Acalles maraoensis + Acalles monasterialis*, etc.) [46]. Such cases were not observed in our data. The large degree of overlap between inter-GD and intra-GD in Entiminae implies that there is no universal threshold at the subfamily level, because the variability of several species was unexpectedly high or low. For example, the maximum intra-GD of *Otiorhynchus armadillo* was 15.74%, whereas the minimum inter-GD of *Polydrusus corruscus* and *Polydrusus impressifrons* was only 0.77%. Nevertheless, we found that the threshold of 9.18% was useful for most species’ delimitation, although it was still too low for *Barypeithes* *pellucidus*, *Otiorhynchus* *raucus*, *Cathormiocerus spinosus*, *Diaprepes abbreviates*, and *Trichalophus tibetanus*. We finally determined the empirical thresholds for species delimitation within 14 genera. For instance, we found higher functional thresholds for species delimitation within the two genera *Barypeithes* (13.48%) and *Otiorhynchus* (13.30%) can delimit the two species *Barypeithes pellucidus and Otiorhynchus raucus*.

Standard thresholds have been investigated in many insect groups. For ladybirds and cryptorhynchine weevils, 3% can be used as the standard threshold for COI barcoding [43,46], 3.3% for pentatomomorphans [18], 5% for fruit flies [50], and a threshold value above 7% for species of *Cleus* [21]. 2.20% is the universal threshold applied in BOLD, and a new OTU (Operational Taxonomic Unit) is formed when the threshold of sample sequence is more than double the standard (>4.40%) [38]. However, these thresholds are found to be too low for Entiminae, 49 of 117 (42%) tested species’ maximum intra-GD were greater than 2.20%, 36 (31%) greater than 3%, 35 (30%) greater than 3.3%, 24 (21%) greater than 5%, and 14 (12%) greater than 7%. Hansen et al. analyzed 41 Entiminae species (70 sequences) in South Africa and detected the maximum intra-GD of 9.20% in *Phlyctinus xerophilus* [4]. The threshold of 9.18% was higher compared to those previously proposed for the delimitation of other taxa. However, we determined this best-fitting threshold through joint analysis of local minima and the maximum intra-GD and minimum inter-GD of each species. If a smaller threshold was applied, the expected result would be an overestimation of species diversity. Large-scale species sampling is usually related to high intra-GD. For example, the maximum K2P intra-GD from 791 individuals belonging to 217 species of Cryptorhynchinae is 21.9% [46], while in Hendrich’s central European Coleoptera DNA barcode reference, the maximum intra-GD of *Leptacinus intermedius* (Coleoptera: Staphylinidae) is 26.03% [42]. A key initial first step should be using empirical data to look for barcoding gaps and threshold values. Future research on Entiminae may benefit from these empirical values as they are more reliable than arbitrary, fixed thresholds. As new sequences and taxonomic knowledge amass, re-evaluating threshold values will improve their accuracy for species delimitation and hence applicability [4,18].

### 4.3. High Intraspecific Divergence Versus Cryptic Species Diversity

High intra-GD may be the result of cryptic species, possibly generated by recent geographical isolation [51,52]. The maximum intra-GD of ten species (*Barypeithes pellucidus*—12.16%, *Cathormiocerus spinosus*—15.80%, *Diaprepes abbreviatus*—12.66%, *Otiorhynchus armadillo*—15.74%, *Otiorhynchus coecus*—16.17%, *Otiorhynchus raucus*—12.27%, *Otiorhynchus tenebricosus*—17.34%, *Polydrusus impressifrons*—18.01%, *Trichalophus caudiculatus*—14.36%, *Trichalophus tibetanus*—14.49%) were higher than the optimal threshold of 9.18% and their sequences were split into 2–13 BINs. A total of 36 specimens of *Diaprepes abbreviatus* were divided into 13 BINs with a maximum intra-GD of 12.66% (JF302960–JF302917). Among these sequences, 13 sequences collected from Dominica were allocated to six unique BINs. Seven sequences collected from Dominican Republic, 15 sequences collected from Puerto Rico, and one sequence collected from the United States, shared a BIN (AAZ7568). The former two additional possessed unique BINs. If the thresholds inferred are truly reflective of species-level diversity, this suggests that there could be more than ten cryptic species within the *D. abbreviatus* species complex. Ascunce et al. amplified COI barcode sequences of *D. abbreviatus* from eggs, larvae, and adults collected from the United States for molecular identification, obtaining three COI haplotypes [53]. Similarly, in species *Otiorhynchus tenebricosus*, three sequences collected from Germany and two sequences collected from Austria were allocated to two different BINs in BOLD, and the maximum intra-GD (17.34%) was detected from the sequence (KM45174–KU91158, KM45174–KU90986) collected from these two countries. There are roughly 1500 validated species in the Palaearctic region alone for this hyper-diverse genus, with many cryptic species waiting to be discovered [54]. Unfortunately, reviewing the morphological characters of the aforementioned species is beyond the scope of the current study.

On the one hand, DNA-based methods help to effectively circumvent the problems caused by traditional morphological taxonomic methods, e.g., incorrect species assignment and confusion between sibling taxa or cryptic species not easily distinguished via morphology [55]. On the other hand, species identification via DNA barcoding should be treated as molecular parataxonomy [56], because DNA barcoding focuses more on species delimitation than on species description [46]. As a reference point, a guiding classification based on morphological characteristics is also necessary [21,57,58]. Although COI-based DNA barcodes have proven successful in delimiting Entiminae species, such an approach based on a single locus can be misled in certain complex situations [59,60]. In view of this, it is highly recommended to combine other types of evidence (e.g., morphological characters, geographic distributions, ecological factors, and reproductive isolation) with molecular information (e.g., mitochondrial and nuclear sequences) to avoid single-gene pitfalls and to better illuminate intra- and interspecific relationships of Entiminae species [61,62,63,64]. With our extensive collection of the current DNA barcodes in Entiminae species, this study is a beneficial exploration of Entiminae species delimitation. However, current resources remain limited, demonstrating the urgent need for a molecular reference library with extensive sampling, high quality sequences, and detailed information such as imaging, geographic coordinates, and more [58]. DNA barcoding identification remain valuable in providing a standardized, web-based delivery and cost-effective solution to the current problem of species identification.

## 5. Conclusions

This study estimated the genetic distance between species within the subfamily Entiminae, based on the public and newly generated COI sequences. We found that there was no universal threshold of genetic distance for all genera for species delimitation. Commonly used thresholds between 2% and 5% were too small to be applied in Entiminae. A threshold of 9.18% was useful for most species at the subfamily level, but thresholds varied by genera. We expect to increase the geographic coverage of samples and refine the taxa at genus level when determining thresholds for species delimitation.

## Figures and Tables

**Table 1:** Sequences of COI primers.

Primer Name	Primer Sequence (5’ to 3’)	Reference	Notes
C_LepFolF (LepF1:LCO1490)	LCO1490 GGTCAACAAATCATAAAGATATTGG LepF1 ATTCAACCAATCATAAAGATATTGG	Hernández-Triana et al., 2014	Cocktail Primer
C_LepFolR (LepR1:HCO2198)	HCO2198 TAAACTTCAGGGTGACCAAAAAATCA LepR1 TAAACTTCTGGATGTCCAAAAAATCA	Hernández-Triana et al., 2014	Cocktail Primer
MLepF1	GCTTTCCCACGAATAAATAATA	Hajibabaei et al., 2006	
MLepR2	GTTCAWCCWGTWCCWGCYCCATTTTC	Hajibabaei et al., 2006	

**Table 2:** Primer sets used to amplify COI barcode sequences of Entiminae.

Primer Set	Length	PCR Success Rate
C_LepFolF + C_LepFolR	658bp	61.1% (33/54)
C_LepFolF + MLepR2	307bp	71.4% (15/21)
MLepF1 + C_LepFolR	407bp	81.0% (17/21)

**Table 3:** Nucleotide composition in the 648 COI barcoding sequences.

Nucleotide Position	Base Number (bp)	Conserved Site	Variable Sites	Parsim–Infor Sites	Singleton Sites	T (%)	C (%)	A (%)	G (%)	AT (%)	CG (%)
The first position	219	97	122	108	14	24.0	18.0	30.5	27.6	54.5	45.6
The second position	219	158	61	50	11	42.7	25.5	15.1	16.7	57.8	42.2
The third position	220	0	220	220	0	39.4	13.4	43.1	4.2	82.5	17.6
All	658	255	403	378	25	35.4	18.9	29.6	16.1	65.0	35.0

## Data Availability

All data generated or analyzed during this study are included in this published article.

## Supplementary Materials

The following are available online at https://www.mdpi.com/article/10.3390/insects13030261/s1, Table S1: Records with COI-5P sequences in BOLD, Table S2: Preliminary screening sequence and species, Table S3: Sequences of Dataset I, Table S4: Specimens information of sequencing data, Table S5: Sequences of Dataset II, Table S6: Intraspecific and interspecific genetic distance, distance threshold for species delimitation, Table S7: The maximum intraspecific and minimum interspecific genetic distances of Entiminae species in Dataset I, Table S8: The maximum intraspecific and minimum interspecific genetic distances of Entiminae species in Dataset II, Table S9: The maximum intraspecific and minimum interspecific genetic distances of Entiminae species in Dataset III, Table S10: The distance threshold for 14 selected genera in Dataset III, Table S11: Primers of COI-5’ sequence of weevils in BOLD database.
